# Editorial: Cognitive and motor skills in sports

**DOI:** 10.3389/fpsyg.2025.1629218

**Published:** 2025-07-14

**Authors:** Sabine Schaefer, Karen Zentgraf, Kylie Ann Steel

**Affiliations:** ^1^Institute for Sport Science, Saarland University, Saarbrücken, Germany; ^2^Work Unit Movement Science and Training in Sports, Institute for Sports Sciences, Goethe University Frankfurt, Frankfurt, Germany; ^3^School of Health Science, Western Sydney University, Sydney, NSW, Australia; ^4^MARCS Institute, Western Sydney University, Sydney, NSW, Australia; ^5^Translational Health Research Institute, Western Sydney University, Sydney, NSW, Australia

**Keywords:** attention, cognition, decision-making, movement, skill acquisition

Our Research Topic assembles 19 contributions that explore cognition and motor skills in sports. Conceptual issues are addressed in two of these papers including Loland et al. who propose an integrative approach in the formation of sports-specific movement-skill theories. The researchers suggest to first attribute phenomenological descriptions of the primary experiential qualities inherent in the execution of the respective skill. This should be followed by multilevel mechanistic analyses (based on biomechanics, motor control approaches, expertise studies, and cognitive science), and culminating in the systematization of findings and the formulation of sport-specific motor skills theories.

Huesmann and Loffing, with attention toward anticipation research, propose a framework to guide study design and reporting. Their paper details a first proposal for a 7-level classification of perception-action coupling conditions, with the defining dimensions of stimulus presentation and response mode orientation. The authors also provide the findings from a review of anticipation and racquet sport studies utilizing the classification system as a template for experimental protocol analysis, revealing underrepresentation of representative perception-action coupling conditions.

Seven contributions to this Research Topic address cognitive-motor dual-task situations across a diverse range of sports. Firstly, Wu et al. provide a review on the effects of cognitive-motor dual-task training, based on 10 acute and 7 chronic studies. For acute effects, studies consistently show performance deteriorations in dual- as compared to single-task situations. Conversely, studies exploring chronic effects show that systematic training in cognitive-motor dual tasking improves performances in cognitive-motor dual-tasks. Montalt-Garcia et al. provide depth to the Canadian Agility and Movement Skill Assessment (CAMSA) with cognitive challenges, resulting in six performance profiles in a large sample of secondary school students. The paper by Monz et al. combines ergometer rowing and Taekwondo exercises with an episodic memory task, revealing pronounced performance decrements in both cognition and motor functioning in the dual-task condition, across different age and expertise levels. Knöbel and Lautenbach tested soccer players with a 3-back working-memory task, either performed on a computer, or with a soccer-specific motor response (shooting toward a specific target location in space). The study reports a significant correlation between performances (response time and accuracy) in the two settings, indicating that the task may be a suitable diagnostic tool for soccer performance.

Klotzbier and Schott a continued the contributions that address cognitive-motor dual-task situations by asking soccer players to perform the classic Trail-Making test while also exposing them to modified versions of the test including movement through space (Trail-Walking test) or dribbling a soccer ball (Trail-Dribbling test). For the Trail-Dribbling test, the authors report shorter test durations for high-level compared to low-level players, with increased cognitive load accentuating differences. The authors conclude that the Trail-Dribbling test allows for an effective discrimination between high and low-level players in the age range of 14 to 17 years. Utilizing 322 elite athletes (ice hockey, closed skill sports, other team sports), Brinkbäumer et al. had subjects perform a tapping task, a visuo-verbal speed-reading task, and both tasks simultaneously. Dual-task costs were found for all sport groups, and costs were more pronounced in closed-skill athletes. For athletes from team sports and ice hockey, the authors did not find a relationship of dual-task costs to performance level. Amara et al. continued this dual task theme and assessed a mental rotation task with and without balance exercises in badminton and volleyball players. Unlike the other studies in the current Research Topic, which consistently found performance deteriorations under dual-task conditions, the reaction times of participants in the Amara et al. study were reduced when balancing concurrently.

An additional sub-theme to emerge amongst the papers submit to this Research Topic was the role of sensory input for sport skills with five submissions. Müller et al. manipulated visual input with stroboscopic glasses in a within-subjects design in Australian Rules football athletes, interrupting the perception-action cycle while kicking the ball at a goal. Interestingly, their study found no performance decrements in kicking accuracy. Continuing with visual factors, Nakazato et al. used an eye tracking device in a virtual table tennis environment with different types of ball trajectories, courses, and speeds. Their findings demonstrate that experienced table tennis players demonstrated lower mean and inter-trial variability in saccade endpoint error compared to novices, which may be indicative of more efficient identification of relevant stimuli. Nicklas et al. introduced a particularly novel study and assessed the role of visual fixations for interpersonal communication in elite sports. Eighteen expert beach volleyball players were exposed to game-like scenarios with high and low performance pressure. They found that higher pressure leads to more and longer fixations on teammates' faces, reflecting a higher need for communication without misunderstandings. In contrast to previous study's Rodrigues et al. explored the gaze behaviors of coaches rather than athletes. The authors showed differences in gaze durations in expert and novice coaches in a variety of game situations for videotaped futsal set pieces. Finally, Kassem et al. tested the eye movements relative to decision accuracy of elite junior Australian Rules football players with 14 brief video clips in two testing sessions in an 18-month time-interval. Participants with accurate decisions responded faster, and skilled participants demonstrated fewer fixations with shorter durations. The authors argue that the task may be a viable tool for Talent Identification and Development.

Our Research Topic also includes studies on embodiment, motor imagery, mental rotation, and skill learning. Luis del-Campo et al. assessed embodied planning in climbing and showed that the handholds where gaze was directed to during pre-planning were used more often than others. Further, experienced climbers ascend faster and look at non-used handholds for a shorter time compared to lesser-skilled climbers. For motor imagery, Tien and Chang re-examine the commonality and distinguishable aspects of motor imagery and execution via a response repetition paradigm. Their results show that motor representations of imagery and execution, when measured without subjective judgments, appear to be more distinguishable than traditionally thought. While Çiftçi and Yilmaz investigated action observation and motor imagery in an intervention study. For drop jump performance, an 8-week-intervention program with motor imagery sessions during video observation did not lead to improvements in physical performance, but there was a positive influence on athlete's perception of their performance. Klotzbier and Schott tested novice and experienced gymnasts and soccer players with different perceptual task (recognition of soccer-specific poses) and with mental rotation tasks using different stimuli (soccer-specific poses, cubes, line-drawings of hands, letters). Their results suggest that gymnasts' motor expertise plays a role in their performance on mental rotation tasks involving both egocentric and object-based transformations, regardless of the stimuli presented. For learning a three-ball juggling cascade, Geller et al. compare a “learning-in-parts” training regime (gradually increasing difficulty) and elements of the juggling movement to an “all-at-once” regime (training on the complete skill from the start). They report initial advantages of the all-at-once group, but no difference in performance between the groups at the end of the training sessions.

This Research Topic thereby enables valuable, current, conceptual as well as empirical insights into cognitive-motor research.

